# Interagency collaboration in primary mental health care: lessons from the Partners in Recovery program

**DOI:** 10.1186/s13033-019-0297-4

**Published:** 2019-05-31

**Authors:** Julie Henderson, Sara Javanparast, Fran Baum, Toby Freeman, Jeffery Fuller, Anna Ziersch, Tamara Mackean

**Affiliations:** 1 Southgate Institute for Health, Society, and Equity, Flinders University, GPO Box 2100, Adelaide, 5001 Australia; 2 College of Nursing and Health Sciences, Flinders University, Adelaide, Australia

**Keywords:** Mental illness, Care coordination, Interagency collaboration, Partners in recovery, Australia

## Abstract

**Background:**

Collaborative care is a means of improving outcomes particularly for people with complex needs. The Partners in Recovery (PIR) program, established in Australia in 2012, provides care coordination to facilitate access to health and social support services for people with severe and persistent mental illness. Of the 48 PIR programs across Australia, 35 were led by Medicare Locals, the previous Australian regional primary health care organisation and nine involved Medicare Locals as partner organisations.

**Aims:**

To identify features which enabled and hindered collaboration in PIR programs involving Medicare Locals and determine what can be learnt about delivering care to this population.

**Methods:**

Data were collected from 50 interviews with senior staff at Medicare Locals and from eight focus groups with 51 mental health stakeholders in different Australian jurisdictions.

**Results:**

Successful PIR programs were based upon effective collaboration. Collaboration was facilitated by dedicated funding, a shared understanding of PIR aims, joint planning, effective network management, mutual respect and effective communication. Collaboration was also enhanced by the local knowledge and population health planning functions of Medicare Locals. Jurisdictional boundaries and funding discontinuity were the primary barriers to collaboration.

## Background

Interagency collaboration is seen as a means of improving primary mental health care. This is because it may improve access to services and reduce service gaps [1]; reduce health care costs through making use of existing resources and reducing service duplication [2], and improve equity in service provision [3]. Such collaboration is also understood as improving the quality of life, and health and well-being of people with complex needs [4]. The use of collaborative service delivery is pertinent to people with severe and persistent mental illness who require a range of health and social supports to maintain social functioning but who frequently experience difficulties in accessing these services [5, 6]. A diagnosis of mental illness is often accompanied by stigma leading to reliance upon a smaller number of people, often family and close friends, who provide informal support through managing aspects of care [7, 8]. In the absence of these networks, people with severe and persistent mental illness often become increasingly dependent upon formal support networks for both treatment and social support [9].

This paper examines the Partners in Recovery (PIR) program which was established in Australia in 2012 to coordinate service delivery for people with severe and persistent mental illness [10]. The Australian Government has a history of supporting interprofessional and interagency cooperation as means of improving primary mental health care [11, 12]. Partners in Recovery (PIR)—funded by the Australian Government—focused on providing care coordination and support to access health and social support services to individuals with severe and persistent mental illness [10]. Of the 48 PIR programs operating in May 2015, 35 had Medicare Locals as a lead organization, and nine PIR programs had Medicare Locals as partner organizations. Four PIR programs were independent of Medicare Locals [13]. Medicare Locals were regional primary health care organizations that were established by the Australian Government to improve coordination of primary health care, address service gaps and improve patient navigation of local health services [14]. Medicare Locals were subsequently replaced by 31 Primary Health Networks (PHNs) in July 2015. PHNs are funded to promote care for the population with severe and persistent mental illness primarily provided through the mental health nurses employed in general practice under the Mental Health Nurse Incentive Program. Social services for people with serious and persistent mental illness that were delivered through PIR are currently being transitioned to the National Disability Insurance Scheme (NDIS) which provides support services for people with ongoing impairment.

PIR was framed around two perceived problems: gaps in mental health services and the challenge for people with severe and persistent mental illness of accessing suitable services [10]. Under PIR, funding was provided to employ support facilitators to broker the services that this population needs [5]. Service delivery was underpinned by service coordination promoted through consortia of mental health service providers and organisations. The stated purpose of the consortia were to map service gaps, ‘join up sectors’ and create ‘collective ownership’ of service delivery by all partners in the consortium [10, 15].

### Successful collaboration in mental health

Valentijn et al. [16] developed a taxonomy for evaluating integrated care focusing upon macro, meso and micro integration. Macro integration explores systemic readiness for integration and involves vertical integration through partnerships between different levels within the health care system e.g.: primary and tertiary health care services and horizontal integration through adoption of a holistic approach to health involving intersectoral partnership [17]. Meso integration occurs at an organizational level and involves interprofessional and inter-organizational integration [16]. Micro integration refers to clinical integration or the “extent to which patient care services are coordinated across various professional, institutional and sectorial boundaries in a system” (17 (p. 7)). Service integration is promoted by functional integration which involves integration of service functions and activities and normative integration which refers to a common frame of reference and shared mission goals. The discussion that follows identifies factors which have been associated with macro, meso and micro integration.

#### Macro integration: systemic factors

People experiencing severe and persistent mental illness require “a system-wide approach” involving partnerships between primary care; state-funded secondary and tertiary mental health services; non-government organizations; and police and emergency services [5]. Systemic factors affecting capacity to support coordinated care include the availability of funding and government mandates about service provision as well as the size and composition of the community to be serviced and pre-existing mechanisms for sharing clients between services [18]. A coordinated approach has traditionally been difficult to achieve in Australia due to the division of responsibility for primary health care services (e.g. GP and Psychology Services) and secondary and tertiary mental health services between federal and state governments respectively [5]. The Australian (federal) Government supports private fee-for-service general practices through Medicare rebates with an increasing out-of-pocket contribution by patients [19] while secondary and tertiary mental health service delivery is a state government responsibility, funded by both state and federal governments. People with severe and persistent mental health conditions frequently require community mental health support services funded by federal, state, and charitable funding. The range of funding options and accountabilities make the system difficult to negotiate for service providers and navigate for users of services [10]. Service ‘silos’, short term funding for support services and the workload of service providers are also implicated [20]. Further, there are limited specialist services to draw upon in outer metropolitan and rural and remote regions [21].

#### Meso integration: organizational factors

Organizational factors also affect collaboration in mental health. Valentijn et al. [16] explore both inter-organizational and interprofessional integration. They argue that the delivery of primary care often occurs via networks. Successful networks require governance strategies that align the participating organizations. Palinkas et al. [18] identify shared service values and a common understanding of the problem; establishment of effective communication between agencies; a clearly defined and equitable division of labour; supportive leadership and development of relationships between key personnel in the participating agencies as important for successful collaboration. Effective leadership is also essential for collaboration and can legitimate collaborative activities, ensure accountability and influence strategy [22]. The effectiveness of complex network depends upon a coordinating group with a clear mandate and sufficient power to implement strategies [23]. Effective leaders have strong interpersonal and organizational skills, a capacity to facilitate team building, retain a focus upon goals and utilise members’ skills in meeting these goals and fairness in dealings with others [24]. Perrault et al. [25] highlight a need for mutual respect as well as recognition of the interests of the participating organizations and the constraints they face in delivering services. They also identify the importance of relationship building between agencies. Relationship building is facilitated in the establishment stage by the development of joint goals and mission statements and in the development stage by frequent communication [2]. Good communication is not only regular but is also clear, transparent and direct [3]. Both formal and informal communication are viewed as promoting relationship building [25].

A second component of meso integration is interprofessional integration [16]. Interprofessional practice is important in the provision of mental health care but networks can inhibit good care through sharing of accountability and hence diffuse responsibilities as well as diverging understandings of appropriate care [17]. Valentijn et al. [17] argue that these issues can be overcome through clear role definitions, communication and mutual respect.

#### Micro integration: clinical integration

A third form of integration is micro or clinical integration to ensure a seamless patient journey through the health care system. For Valentijn et al. [17] clinical integration should be person rather than disease focussed with services designed to be holistic and to meet the needs identified by patients. Clinical integration depends upon the processes established to promote interagency collaboration. Fuller et al. [22] in a review of collaborative strategies used in mental health, identify four types of strategies that can be used to link organizations. These are: direct collaborative activities; agreed guidelines; effective communication systems; and formalized service agreements. They found that collaboration was strongest when all four elements were present. For Brophy et al. [5] the appointment of a network manager to promote negotiation, to act as a ‘policy entrepreneur’ and to span service boundaries may also facilitate effective service implementation.

This paper adds to the above insights on collaboration by reporting research identifying features of successful collaboration in the PIR programs involving Medicare Locals. It then derives lessons learnt from PIR about how to best support people living with mental illness. This research is timely as the transition of many PIR functions to the National Disability Insurance Scheme (NDIS) may adversely affect access to and the level of social support available for people with severe and persistent mental illness.

## Methods

### Data collection

Data were collected as part of a larger NHMRC project examining population health planning in regional primary health care organizations in Australia, particularly for three population groups: Aboriginal and Torres Strait Islanders, migrants and refugees, and people with mental illnesses. The project collected and analysed secondary and primary data from Medicare Locals including review of planning documents, online survey and individual interviews, and stakeholders’ focus groups. Data for this paper were drawn from (1) mental health stakeholder focus groups, and (2) Medicare Local senior executive and board member interviews.

#### Mental health stakeholder focus groups consultations

Mental health stakeholder focus group consultations were held in each State and Territory (n = 8) between April and June 2015 with 51 people representing carer and consumer organizations, NGOs and service providers who had worked with Medicare Locals in delivering mental health services attending (see Table 1). Participants were recruited through a list of relevant community and mental health organisations developed in each State and Territory. An invitation was sent to the CEOs of these organisations to seek permission and request 1 or 2 people from their organization to attend the consultation session. The consultations were facilitated by two members of the research team, and focused on experiences of working with Medicare Locals and state services in the delivery of mental health care and recommendations for ways of improving partnerships in regional primary health care organizations.

**Table 1:** Number and organizational background of participants involved in stakeholder consultations

State/Territory	No. of attendees	Organisational background
New South Wales	6	Carer and consumer groups NGO service providers
Victoria	8	Carer and consumer groups NGO service providers
Australian Capital Territory	2	Carer and consumer groups
Northern Territory	10	Carer and consumer groups NGO service providers Territory mental health services
South Australia	6	Carer and consumer groups NGO service providers
Tasmania	13	Carer and consumer groups NGO service providers State mental health services Private practitioner
Queensland	6	Carer and consumer groups NGO service providers State mental health services
Western Australia	7	NGO service providers State mental health services

#### Interviews with senior executives (including CEOs) and board members of Medicare Locals

Phone interviews with 51 senior executives and board members of the Medicare Locals were conducted between October 2014 and January 2015 (see Table 2). Participants were recruited from Medicare Local staff who had undertaken an online survey as part of this study (email distributed to all Medicare Locals in September–November 2014). A total of 210 survey responses were received from 52 Medicare Locals, of which 106 (50%) indicated their willingness to participate in a follow up interview. Of these, 51 were invited to participate in an interview, purposively selected on the basis of seniority, involvement in population health planning, and to maximise geographic spread (by state/territory, and by metro/rural/remote). One person had changed role and declined, resulting in 50 interviews conducted. The interviews were conducted by phone by a member of the research team, and addressed the role of population health planning in Medicare Locals and the capacity of Medicare Locals to address health inequities. People with mental illness were identified as a population experiencing health inequities and the interview guide contained specific questions seeking information about mental health planning by Medicare Locals, and their partnership with mental health organizations in planning and programs implementation.

**Table 2:** Number and role of people involved in Medicare Local interviews

State/Territory	No. of people interviewed	Role
New South Wales	13	12 senior executives 1 Board member
Victoria	9	8 senior executives 1 Board member
Australian Capital Territory	1	1 Board member
Northern Territory	1	1 Board member
South Australia	9	9 senior executives
Tasmania	4	3 senior executives 1 Board member
Queensland	11	10 senior executives 1 Board member
Western Australia	3	3 senior executives

### Analysis

All interviews and focus group sessions were audiotaped and transcribed verbatim and transcripts were analysed deductively and inductively. The initial framework for thematic analysis was provided by the interview schedules with additional themes added upon review of the transcripts [26]. The theoretical framework was applied after initial review of the data revealed enablers and barriers related to systemic, organisational and process factors. Data were coded by two people working independently of each other and managed in NVivo11.

### Ethics, consent and permission

Ethics approval to conduct the mental health stakeholder focus groups and key informant interviews with senior staff at the Medicare Locals was obtained through the Flinders University Social and Behavioral Research Ethics Committee. Written consent was obtained from all participants in the focus group and interviews prior to data collection and participants were informed via an information sheet that data may be used in publications.

## Results

The PIR program was generally viewed favorably by both Medicare Local participants and key stakeholders, with the successes or failures of the PIR program in any particular jurisdiction largely attributed to the extent and nature of collaboration between the organizations in the consortium. The discussion that follows outlines the factors which enabled or hindered the delivery of integrated mental health care through PIR in consortia involving Medicare Locals. Data are drawn from respondents’ experiences of collaboration and from questions about how they would like to see PHNs approach primary mental health care. Following Valentijn et al. [16], the results are presented through the lens of macro, meso and micro integration. The results are summarized in Table 3.

**Table 3:** Summary of findings

	Barriers	Enablers
Macro integration	Jurisdictional boundaries (intergovernmental and intersectoral) Sustainability and amount of funding Meeting funding requirements Service siloing	Dedicated funding
Meso integration	Lack of agreement about the focus of care Failure to recognize the expertise of service providers	Knowledge of mental health Sharing of information Respect for service providers Local knowledge Population health planning identifying distribution and gaps in mental health services
Micro integration	Centralisation of management once funding was obtained	Joint planning Designated PIR managers with program oversight Development of relationships between service managers Centralized intake Shared electronic records

### Macro integration: systemic factors

The external environment was generally viewed as a barrier to successful delivery of collaborative mental health care with jurisdictional boundaries and funding particularly highlighted. Successful community support for people with serious mental illness involves an inter-sectoral approach utilising both clinical and social services [27]. The need for an intersectoral approach was noted by some participants. A participant in the NSW focus group, for example, stated that:

*I think they need to look outside of the clinical sphere to get the solutions. …it takes a whole system so we work across housing providers, income training, skills development, legal, everything physical as well as mental health services* (NSW focus group).

Participants identified barriers arising from poor coordination across levels of government, intersectorally and across state and territory boundaries. Participants noted that state governments responded to federal spending on mental health by withdrawing services. This approach reduced service duplication but creates “*disconnects between State funded activity and Commonwealth funded activity”* (Senior Executive, Vic). Coordination of care was also inhibited by competing policy agendas and different approaches to service delivery resulting in lack of horizontal integration. A respondent from the Queensland stakeholder focus group noted that siloing negatively affects collaboration.

*[The purpose of the] whole PIR program is not to work in silos. And it appears that they do. So there’s primary health and there’s mental health and then there’s LGBTI, you know, Aboriginal and Torres Strait Islander* (Qld focus group).

Dedicated funding was viewed as enabling collaboration. A respondent from a Queensland Medicare Local noted that funding for mental health is usually complex with “*different funding programs at a State and Commonwealth level*” (CEO, Queensland). The allocation of dedicated funding for PIR brought competing agencies “*together around one pool of funding where they’ve decided what they want to do*” (CEO, Queensland). Conversely, structural change in the form of the restructuring of Medicare Locals into PHNs was accompanied by concerns about whether funding for some social support services would continue. PHNs have received additional funding to provide mental health services however that funding is currently tied to existing clinical programs such as ‘headspace’ and the Access to Allied Psychological Services (ATAPS) program both of which primarily address early intervention [28]. This change was associated with concerns about the sustainability and amount of funding for existing programs and the impact that funding would have on service continuity. A participant from Tasmania in discussing the transition of the program noted that:

*There was never long term commitment though because they changed governments, and mostly you’ll get something for five years, and that’s if you’re lucky, and then a new government comes in and says, “Oh, no, we don’t need to spend money in that area.” That gets wiped* (Tasmanian focus group).

Lack of flexibility in how funding was to be used was identified by some participants. This concern has also been noted by Supper et al. [29] who say that flexibility is important in responding to the changing needs of people with mental illness. A respondent from the Northern Territory stated:

*….with the Partners in Recovery Program…they had NGOs providing the service but at the end of the day they were telling the NGOs what they could and couldn’t do and they were very descriptive [prescriptive] around what that looked like* (NT focus group).

Other PIR consortia reported using any discretion available to provide the services that they felt were needed. A Queensland executive reported using PIR funding to provide mental health support to migrant communities through a local multicultural organization as an example.

### Meso integration

The participant organisations’ characteristics were generally viewed as an enabler to collaboration. The establishment of PIR was generally viewed positively by stakeholders as “*[i]t’s made the Medicare Locals have to actually work with different agencies that they probably haven’t really engaged with traditionally* (Vic focus group). This was viewed as a means of breaking down service silos and creating common goals for service delivery.

*The Partners in Recovery people actually sit in the NGOs and then they connect people to the different services. So it breaks down those boundaries of patch protection and all those things, that consortium model and you’ve got to work out your differences and you’ve got to deal with whatever is happening* (SA focus group).

Successful collaborations were based on a shared understanding of the problem and clear guidelines for the role of the consortia. A senior executive from a Victorian Medicare Local highlighted the need for “*explaining what the criteria were and what the job was, what we had to do*” when establishing networks for PIR. A feature of successful PIR programs was population planning to identify service need and existing mental health resources. This was viewed as an advantage of working with Medicare Locals. A South Australian respondent noted, that “*service mapping was a part of that program so there was some sense of looking at where populations were and where needs were and where services currently are located*” (SA focus group). Similar claims were made by a senior executive from Victoria who stated:

*….population and health planning were critical in that sense, giving us the data about where people were, specifically things like boarding houses…, the jails, the justice system, all those sorts of things where typically those clients would be, so the target areas were fairly well*-*defined for us* (Senior Executive, Victoria).

Successful networks were also based on mutual respect. A respondent from the Queensland focus group when asked what PHNs could learn from PIR stated:

*I think we’ve all identified starting with the premise of respect, trust, a collaborative approach, a commitment to do what you say you’re going to do, a clear transparent process in working with the sector and working with a variety of sectors and variety of stakeholders* (Qld focus group).

Successful programs also identified and focused upon a common cause and encouraged innovation through investing in other organizations. A respondent from the South Australian focus group noted:

*I think the model that [Medicare Local] put in place was very effective because what it then did also was invest in a whole range of different organisations. So they employed support facilitators and there’s innovation and collaboration grants (SA focus group).*

There was evidence however, of poor interprofessional integration. Difficulties arose when partners felt that their views were devalued or not considered. Some respondents associated lack of respect with tensions between clinical and social services. A respondent from NSW focus group viewed some staff from Medicare Locals as incorrectly treating community organizations as the “*Benny Hill mob*”^1^ with limited understanding of mental health.

The employment of staff by Medicare Locals who had previous mental health experience and knowledge of local services was also seen as facilitating collaboration. A Queensland respondent highlighted the role of senior staff who had worked in mental health services in the success of one PIR program. Likewise, a Western Australian respondent associated success with the employment of:

*….people with specific mental health skills and also an understanding of that particular geographical area where that particular PIRs been set up* (WA senior executive).

The employment of people with lived experience of mental health is also important. A senior executive from NSW identifies the role played by *“a consumer consultant who has lived experience of mental illness and he will support consumers and provide information and presentations and education*.” The employment of staff with mental health experience was identified as more difficult for rural and remote regions as suitably trained staff were more difficult to recruit and retain.

### Micro integration: clinical integration

Micro integration is concerned with service seamlessness. While respondents from Medicare Locals view PIR as successful there is evidence from stakeholder interviews that results are mixed. A respondent from the Western Australian focus group noted that:

*Some of the PIRs are doing a great job and some of them are doing an absolute spin and are an absolute waste of money, because they have that whole space in between hasn’t been clearly described or actually collated* (WA focus group).

Clinical integration is associated with the processes established to promote service integration. A senior executive from a NSW Medicare Local identified strategies adopted by their organization to improve service delivery.

*….we’ve put a number of strategies in place that has improved things probably over the last 12 to 18* *months and they include a centralised intake, electronic medical record. I’ve already talked about moving from a contractor model to an employee model and we have targets and KPIs for our counselling sessions.*

Relationship building is also important to the manner in which partners work together. Relationship building requires time. A senior executive from a South Australian Medicare Local stated that “*To get quality engagement it has to be relationship based and it has to take time to develop*.” Successful PIR programs were built upon good communication. A Queensland respondent in describing a successful PIR program stated that “*the reason for that success has been about the communication, has been about the collaboration*” (Queensland focus group). Communication can occur at several levels. A senior executive from Queensland noted that:

*…we have a general manager who might interact with some of those bodies and a health services general manager and then there’s a mental health manager who might interact with sort of the managers within those groups. So where it’s possible, we try to have up and down through the organisation, appropriate contact if you like because it gives us some kind of consistency in the relationship* (Senior Executive, Queensland).

Another feature that was identified as an essential component for success was joint decision making. Successful programs involved “*joint ownership around the planning process*” (SA focus group). In some cases, participants described the overriding of consortium decisions by the Medicare Local. A respondent from Queensland described a PIR program in which “*you made a decision in the consortia, but it could be something completely different by the time you came back to the next meeting*”. In other cases there was a perception that partners were excluded from decision making once funding had been received. A respondent from Northern Territory stated:

*….we thought we were going to be a consortium for Partners in Recovery, however the Medicare Local then said* - *once they were awarded the funding for the tender they said ‘no, it was a consortium to get the tender’* (NT focus group).

Clinical governance is a fourth feature of successful PIR programs. A feature of successful programs was the designation of a network manager (team leader) who could be based either within the Medicare Local or within partner organisations. Brophy et al. argue that the role of a network manager to build and facilitate relationships between services was distinct from the support facilitator role which is client focussed, and was an important component of successful partnership [5]. A senior manager from New South Wales identified the impact that one of the network managers had in coordinating services.

*They work with their local services to link people in so that our clinicians are aware of the services that are in their local regions and it might be employment services or we’ve got Partners In Recovery that we’re the lead agency for as well so they’re aware of them and they can refer to them* (Senior Executive, NSW).

Where this role was lacking, respondents identified a leadership vacuum in which services developed individual programs and information was not shared.

*There has been nowhere to take information. The idea was that you work with a person and they will identify their needs and you’ll have a sense of perhaps what those barriers are for that person and also feedback from the community around what the barriers are, and you will feed that up, which we’ve been doing, but there is no one to hold that information* (Qld focus group).

## Discussion

This paper has presented data from stakeholder consultations and interviews with senior executives in Medicare Locals about the features of successful collaboration in Partners in Recovery. PIR was established to improve service access for the people with severe and persistent mental illness. Lorant et al. argue that people with mental illness require diversified networks containing both clinical and social services to remain in the community successfully [30]. The bringing together of clinical and social services under the banner of PIR was identified as a strength of the program. Our respondents viewed successful consortia as breaking down silos between social and clinical services and enhancing horizontal integration through creating links between Medicare Locals, general practice and the NGOs providing social services to the mentally ill. The governance of the network is also important. Nicaise et al. argue that integrated community care for people with severe mental illness is best provided by a network with a centralized structure, fewer pathways for patients and a lead organization overseeing services [31]. PIRs were based on a consortium model with the majority of PIRs having a Medicare Local as the lead organization.

Successful collaboration was an important part of successful PIR programs. The allocation of dedicated funding to form consortia enabled joint service planning and overcame difficulties arising from disconnect between Commonwealth and State funding sources. Successful consortia also had a designated network manager to build relationships and facilitate communication. Brophy et al. (p. 399) [5] identified a need for a ‘boundary spanner’ to build PIR networks. Successful PIR programs were identified as having an identified leader who undertook project management functions through ensuring decisions made at meetings were communicated and acted upon.

Successful consortia were based on collaboration and effective collaboration was based upon a shared understanding of the purpose of the collaboration and respect for the views of service providers. PIR consortia were required to respond to guidelines established by the Australian Government in bidding to host PIR and communication of these goals to partner members by the Medicare Locals facilitated relationship development. Dissemination of information about program goals; community consultation upon establishment of the consortia; and transparency and joint decision making once the consortia were established facilitated a shared understanding of service goals and promoted normative integration [17]. A shared understanding of program goals was also facilitated by Medicare Locals by the employment of people with knowledge and experience of working in mental health.

Relationship building and effective communication were also identified as features of successful PIR programs. Communication is facilitated by a common language and the development of relationships between key personnel in the participating agencies [18]. Our participants described successful programs as having links across all levels of the participating organizations and scope for discussion and joint planning by consortia increasing commitment to consortia goals. Milward et al. argue that joint problem solving and dispute resolution also lead to improved performance by mental health networks [32].

Participants were also asked to comment upon the lessons learnt from PIR for primary mental health service delivery in PHNs. One issue raised was service continuity, with concern that the move from service provision to commissioning may cause disruption and changes in what services were funded. PIR was based upon sharing of clients and collaboration between health and social services to improve outcomes for people with severe and persistent mental illness. The focus of primary mental health care in PHNs is upon improving access to clinical services through a stepped care model, with PHNs receiving targeted funding for existing programs such as headspace and psychological services. Clinical services for people with severe and persistent mental illness are the responsibility of psychiatric services and general practice supported by mental health nurses employed through the mental health nurse incentive program to provide day-to-day management of clinical issues [33]. This change was associated by our participants with the potential loss of social services. Social services for people with serious and persistent mental illness that are currently delivered through PIR are transitioning to the NDIS which provides disability services, supplemented by state and Commonwealth funding for people who are ineligible for NDIS services. This separation of responsibility for service delivery between clinical and social services potentially reintroduces the ‘silos’ which were identified as inhibiting care for this group reducing horizontal integration of services [17].

While the NDIS has the capacity to provide individualized care plans based upon client’s identified need, critics have noted that mental illness does not fit comfortably within a disability model as it is often episodic leading to concerns with continued eligibility for social services [34]. Smith-Merry et al. [35] note that on government figures current at 2017 nearly 20% of people with psychosocial disability who request access into NDIS are not accepted. Further, reliance upon Mental Health Nurse Incentive Programs may be problematic as PIR was designed to target a population who traditionally have poor access to services. Reliance upon mental health nurses working within general or psychiatric practices potentially limits service provision to those already seeking medical help. Furthermore, uptake of the mental health nurse incentive program is poor in Western and South Australia and the Northern Territory leading to gaps in service delivery [36].

### Limitations

Data for this paper were drawn from interviews and focus groups exploring the role of population health planning in Medicare Locals. People with mental illness were identified as a priority group and questions were asked about management of mental health in general. As a consequence, participants were not questioned specifically about PIR leading to lack of information about specific programs and difficulties in differentiating on the basis of consortia characteristics. This is rendered less problematic by a focus upon enablers and barriers to integration rather than network type. The methodology also precludes the collection of data about the 4 PIR consortia that do not have Medicare Local involvement which may be quite different.

## Conclusion

This paper has outlined barriers and enablers of successful collaboration in PIR programs involving Medicare Locals. PIR programs used consortia to coordinate care for people with severe and persistent mental illness. PIR consortia were designed to provide coordinated care for people with severe and persistent mental illness who may fall through service gaps due to the complexity of their needs. Participants argued that PIR consortia overcame barriers arising from fragmented responsibility and funding for mental health. Program success was associated with successful collaboration based upon mutual respect, communication and effective network leadership. The involvement of Medicare Locals improved service planning through service mapping and need analysis. The transition from Medicare Locals has been associated with renewed separation of clinical and social care for people with severe and persistent mental illness. We argue that this change may reduce service access for the most vulnerable population through reintroducing the siloing that PIR programs addressed.

## Data Availability

Available on request from authors.

## Abbreviations

PIR: partners in recovery
PHN: Primary Health Network
GP: general practitioner
NHMRC: National Health and Medical Research Council
NGO: non-government organization
ATAPS: Access to Allied Psychological Services

## Footnotes

1 Benny Hill is an English comedian suggesting that this group experienced not being taken seriously.

## References

[1] Petrich M, Ramamurthy V, Hendrie D, Robinson S (2013). Challenges and opportunities for integration in health systems: an Australian perspective. J Integr Care..

[2] Tseng Sh, Liu K, Wang W-L (2011). Moving toward being analytical: a framework to evaluate the impact of influential factors on interagency collaboration. Child Youth Serv Rev.

[3] Cooper M, Evans Y, Pybis J (2016). Interagency collaboration in children and young people’s mental health: a systematic review of outcomes, facilitating factors and inhibiting factors. Child..

[4] Cameron A, Lart R, Bostock L, Coomber C (2014). Factors that promote and hinder joint and integrated working between health and social care services: a review of research literature. Health Soc Care Community.

[5] Brophy L, Hodges C, Halloran K, Grigg M, Swift M (2014). Impact of care coordination on Australia’s mental health service delivery system. Aust Health Rev.

[6] Cummings SM, Kropf NP (2009). Formal and informal support for older adults with severe mental illness. Aging Mental Health..

[7] Martin JK, Pescosolido BA, Tuch SA (2000). Of fear and loathing: the role of ‘disturbing behavior’, labels, and causal attributions in shaping public attitudes toward people with mental illness. J Health Soc Behav.

[8] Perry BL, Pescosolido BA (2012). Social network dynamics and biographical disruption: the case of “first-timers” with mental illness. Am J Sociol.

[9] Crotty MM, Henderson J, Ward PR, Fuller J, Rogers A, Kralik D (2015). Analysis of social networks supporting the self-management of type 2 diabetes for people with mental illness. BMC Health Serv Res.

[10] Smith-Merry J, Gillespie J (2016). Embodying policy-making in mental health: the implementation of Partners in Recovery. Health Sociol Rev..

[11] Banfield MA, Gardner KL, Yen LE, McRae IS, Gillespie JA, Wells RW (2012). Coordination of care in Australian mental health policy. Aust Health Rev..

[12] Collins PY, Insel TR, Chockalingam A, Daar A, Maddox YT (2013). Grand challenges in global mental health: integration in research, policy, and practice. PLoS Med.

[13] Australian Government Department of Health. List of funded PIR organisations. 2015.

[14] Horvath J. Review of Medicare Locals: report to the Minister for Health and Minister for Sport: Department of Health; 2014.

[15] Australian Government Department of Health and Ageing. Partners in Recovery: coordinated support and flexible funding for people with severe and persistent mental illness and complex needs initiative: Program guidelines for the engagement of PIR organisations 2012-13 to 2015-16. 2012.

[16] Valentijn P, Boesveld I, van der Klauw D, Ruwaard D, Struijs J, Molema J, Bruijnzeels M, Vrijhoef H (2015). Towards a taxonomy for integrated care: a mixed-methods study. Int J Integr Care.

[17] Valentijn P, Schepman S, Opheij W, Bruijnzeels M (2013). Understanding integrated care: a comprehensive conceptual framework based on the integrative functions of primary care. Int J Integr Care.

[18] Palinkas LA, Fuentes D, Finno M, Garcia AR, Holloway IW, Chamberlain P (2014). Inter-organizational collaboration in the implementation of evidence-based practices among public agencies serving abused and neglected youth. Adm Policy Ment Health Ment Health Serv Res.

[19] Australian Government Department of Health and Ageing (2010). Building a 21st Century Primary Health Care System: Australia’s First National Primary Health Care Strategy.

[20] Henderson J, Battams S (2011). Mental health and barriers to the achievement of the ‘right to health’. Austr J Prim Health..

[21] Wakerman J, Humphreys JS, Wells R, Kuipers P, Entwistle P, Jones J (2008). Primary health care delivery models in rural and remote Australia—a systematic review. BMC Health Serv Res..

[22] Fuller JD, Perkins D, Parker S, Holdsworth L, Kelly B, Roberts R (2011). Building effective service linkages in primary mental health care: a narrative review part 2. BMC Health Serv Res..

[23] Fleury M (2005). Quebec mental health services networks: models and implementation. Int J Integr Care.

[24] Marek LI, Brock D-JP, Savla J (2015). Evaluating collaboration for effectiveness: conceptualization and measurement. Am J Eval.

[25] Perrault E, McClelland R, Austin C, Sieppert J (2011). Working together in collaborations: successful process factors for community collaboration. Adm Soc Work..

[26] Fereday J, Muir-Cochrane E (2006). Demonstrating rigor using thematic analysis: a hybrid approach of inductive and deductive coding and theme development. Int J Qual Methods..

[27] Bower P, Gilbody S (2005). Managing common mental health disorders in primary care: conceptual models and evidence base. BMJ.

[28] Henderson J, Javanparast S, MacKean T, Freeman T, Baum F, Ziersch A (2018). Commissioning and equity in primary care in Australia: views from Primary Health Networks. Health Soc Care Community.

[29] Supper I, Catala O, Lustman M, Chemla C, Bourgueil Y, Letrilliart L (2015). Interprofessional collaboration in primary health care: a review of facilitators and barriers perceived by involved actors. J Public Health..

[30] Lorant V, Nazroo J, Nicais P (2017). Optimal network for patients with severe mental illness: a social network analysis. Adm Policy Ment Health Ment Health Serv Res..

[31] Nicaise P, Dubois V, Lorant V (2014). Mental health care delivery system reform in Belgium: the challenge of achieving deinstitutionalisation whilst addressing fragmentation of care at the same time. Health Policy.

[32] Milward HB, Provan K, Fish A, Islet K, Huang K (2010). Governance and collaboration: an evolutionary study of two mental health networks. J Public Adm Res Theory..

[33] Department of Health PHN Primary Mental Health Care flexible funding pool implementation guidelines: Primary mental health care services for people with severe mental illness; 2016, http://www.health.gov.au/internet/main/publishing.nsl/content/PHN-Mental_Tools. Accessed 26 Sept 2017.

[34] Nicholas A, Reifels L. Mental Health and the NDIS: a literature review. Mind Australia Ltd; 2014.

[35] Smith-Merry J, Hancock N, Bresnan A, Yen I, Gilroy J, Llewellyn G (2018). Mind the gap: the national disability insurance scheme and psychosocial disability. Final report: stakeholder identified gaps and solutions.

[36] Happell B, Platania-Phung C (2017). Review and analysis of the mental health nurse incentive program. Aust Health Rev..

